# A functional variant at the miRNA binding site in HMGB1 gene is associated with risk of oral squamous cell carcinoma

**DOI:** 10.18632/oncotarget.16120

**Published:** 2017-03-11

**Authors:** Chiao-Wen Lin, Ying-Erh Chou, Chia-Ming Yeh, Shun-Fa Yang, Chun-Yi Chuang, Yu-Fan Liu

**Affiliations:** ^1^ Institute of Oral Sciences, Chung Shan Medical University, Taichung, Taiwan; ^2^ Department of Dentistry, Chung Shan Medical University Hospital, Taichung, Taiwan; ^3^ School of Medicine, Chung Shan Medical University, Taichung, Taiwan; ^4^ Department of Medical Research, Chung Shan Medical University Hospital, Taichung, Taiwan; ^5^ Institute of Medicine, Chung Shan Medical University, Taichung, Taiwan; ^6^ Department of Otolaryngology, Chung Shan Medical University Hospital, Taichung, Taiwan; ^7^ Department of Biomedical Sciences, College of Medicine Sciences and Technology, Chung Shan Medical University, Taichung, Taiwan; ^8^ Division of Allergy, Department of Pediatrics, Chung Shan Medical University Hospital, Taichung, Taiwan

**Keywords:** HMGB1, polymorphism, OSCC, bioinformatics analysis, haplotypes

## Abstract

Oral squamous cell carcinoma (OSCC) is a common malignancy that has been causally associated with both hereditary and acquired factors. The high mobility group box 1 (*HMGB1*) gene plays an important role as a DNA chaperone to help maintain nuclear homeostasis. Altered expression of *HMGB1* has been implicated in a wide range of pathological processes, including inflammation and cancer. The present study explores the impact of *HMGB1* gene polymorphisms, combined with environmental risks regarding susceptibility to oral tumorigenesis. Four single-nucleotide polymorphisms (SNPs) of the *HMGB1* gene, rs1412125, rs2249825, rs1045411, and rs1360485, were evaluated in 1,200 normal controls and 772 patients with OSCC. We found an association between the wild-type allele of rs1045411 and genotypes CT and CT/TT (AOR=0.754, 95% CI=0.582-0.978 and AOR=0.778, 95% CI=0.609-0.995, respectively). Additionally, bioinformatics analysis was used to characterize the functional relevance of these variants for the miRNA-505-5p binding site and transcriptional regulation by the *HMGB1* 3’-UTR and promoter regions. Moreover, in considering behavioral exposure to environmental carcinogens, the presence of the four *HMGB1* SNPs, combined with/without betel quid chewing and smoking showed, profoundly synergistic effects on the risk of OSCC. In conclusion, we present a potential clinical relevance for *HMGB1* variants in OSCC, as well as associations between *HMGB1* polymorphisms, haplotypes and environmental risk factors. The finding may help in development of optimal therapeutic approaches for OSCC patients.

## INTRODUCTION

Oral squamous cell carcinoma (OSCC) is the sixth most common malignancy worldwide, with 599,000 new cases and 325,000 cancer deaths in 2012[1, 2]. OSCC, which accounts for more than 90% of all mouth malignancies and approximately 40% of head and neck tumours has, a high potential for local invasion and lymph node metastasis. Indeed, the estimated 5-year overall survival rate of less than 50% has not improved significantly over the last three decades [3]. OSCC often causes postsurgical dysfunctions in chewing, swallowing, and speech, as well as aesthetics loss [4]. Moreover, although early-stage OSCCs has a high treatment success rate approximately 70% of patients with progressive disease cannot be successfully treated due to relatively high local and regional recurrence rates [5]. Therefore, early detection and clarification of the detailed molecular mechanism of OSCC are urgently needed [6, 7].

The *HMGB1* gene belongs to the high-mobility group protein family, and the protein product contains two 80-amino acid DNA-binding domains (A-box and B-box) and an negatively charged C-terminus [8]. The gene sequence is located on the long arm of chromosome 13; the transcriptional region spans approximately 6,000-bp, with a promoter region of at least 1,700-bp, and 5 exons of approximately 2,600-bp [8]. *HMGB1* functions in the nucleus as a chromatin structural protein and extracellularly as a pro-inflammatory cytokine [9, 10]. Nuclear *HMGB1* is a non-histone DNA-binding protein, which suggests that it facilitates the assembly of site-specific DNA targets by acting as a DNA chaperone [11]. In contrast, extracellular *HMGB1* functions as a damage-associated molecular pattern that propagates infection- or injury-elicited inflammatory responses [12]. Moreover, evidences confirm that *HMGB1* over-expression is closely related to tumour development through functions in proliferation, invasion and migration of cancer cells [13–16]. Therefore, *HMGB1* may serve a biomarker of inflammation and/or a prognostic marker for OSCC progression [9, 17, 18].

Although the development of OSCC may take several decades years, early detection of this cancer is seldom achieved due to the lack of reliable markers [19]. Therefore, the disease runs a largely asymptomatic course until it becomes too advanced for successful treatment [20, 21]. Aberrations in some genes may be responsible for certain clinical features of OSCC [22]. For instance, differences in the level of *HMGB1* expression have been demonstrated between precancerous and malignant lesions [23–25] yet little is known regarding the joint effects of *HMGB1* gene variants and behavioural exposure to cancer-causing substances on predisposition to OSCC. Moreover, previous studies have reported the effect of *HMGB1* gene polymorphisms on human cancer susceptibility, and the polymorphisms may efficiently predict the risk of cancers [26–30]. Thus, we hypothesized that four polymorphisms (rs1412125, rs2249825, rs1045411, and rs1360485; Table 1) in *HMGB1* are associated with susceptibility to OSCC. The aim of this study was to examine the potential clinical relevance of four SNPs in the *HMGB1* gene in patients with OSCC, as well as associations between *HMGB1* polymorphisms, haplotypes and environmental risk factors.

**Table 1:** Variants, positions, function and of high mobility group box 1 (*HMGB1*) sequence variations

	Chromosome position (Chr.13)			
	SNP 1	SNP 2	SNP 3	SNP 4
**Genome position †**	31,041,595	31,037,903	31,033,232	31,031,884
**Nucleotide change**	T>C	C>G	C>T	T>C
**dbSNP (rs number)**	rs1412125	rs2249825	rs1045411	rs1360485
**Molecular consequences ‡**	2KB upstream	intron 1	3’-UTR	500B downstream
**Function prediction**	enhancer	Transcriptional factorbinding site	miRNA binding site	-
**Heterozygous (%) ‖**	44.4	28.0	33.5	36.3

^†^GRCh37.p13

^‡^The Molecular consequences annotated by human *HMGB1* mRNA (NM_002128.5).

^‖^Specific heterozygosity frequencies using the East Asian population (1000 Genomes Project phase 3).

## RESULTS

### Study population

A total of 1,972 participants, including 772 OSCC cases and 1,200 controls were recruited to explore the effects of the *HMGB1* gene on OSCC risk. Demographic characteristics including mean age, betel quid chewing, cigarette smoking and alcohol drinking are shown in Table 2. We discovered significant differences when grouping by betel quid chewing (*P*<0.001), cigarette smoking (*P*<0.001) and alcohol drinking (*P*<0.001) in the healthy controls and OSCC patients (Table 2).

**Table 2:** The distributions of demographical characteristics in 1,200 controls and 772 patients with oral squamous cell carcinoma (OSCC)

Variables	Controls (N=1,200)	Patients (N=772)	*P* value †
**Age (yrs)**	**Mean ± S.D.**	**Mean ± S.D.**	
	53.91 ± 10.02	54.80 ± 11.04	*P=*0.063
**Betel quid chewing**	**n (%)**	**n (%)**	
No	1,001 (83.4%)	154 (19.9%)	***P*<0.001**
Yes	199 (16.6%)	618 (80.1%)	
**Cigarette smoking**			
No	564 (47.0%)	86 (63.2%)	***P*<0.001**
Yes	636 (53.0%)	686 (88.9%)	
**Alcohol drinking**			
No	963 (80.3%)	340 (44.0%)	***P*<0.001**
Yes	237 (19.8%)	432 (56.0%)	

^†^Mann–Whitney U test or Fisher's exact test was used between controls and patients with OSCC.

### *HMGB1* associated polymorphisms and environmental risk factors

Table 3 summarizes the basic characteristics of the *HMGB1* SNPs in the study population. In both the OSCC patients and healthy control subjects, genotypes T/T, C/C, C/C, and T/T exhibited the highest frequencies for rs1412125, rs2249825, rs1045411 and rs1360485, respectively. In these controls, the genotypic frequency of *HMGB1* SNP rs1412125 met the Hardy-Weinberg equilibrium (*P*=0.282, χ2 value: 1.159). The frequencies of *HMGB1* SNPs rs2249825, rs1045411 and rs1360485 were also in the Hardy-Weinberg equilibrium (*P*=0.678, χ2 value: 0.172; *P*=0.451, χ2 value: 0.569; and *P*=0.537, χ2 value: 0.382, respectively). According to the adjusted odds ratios (AORs) and 95% confidence intervals (CIs) in a multiple logistic regression model for *HMGB1* polymorphism and OSCC, compared with their corresponding wild-type homozygotes (C/C), only rs1045411 C/T or C/T+T/T presented a significant (*P<*0.05) protective role after adjusting confounding factors: 0.754-fold (95% CI, 0.582-0.978) and 0.778-fold (95% CI, 0.609-0.995), respectively (Table 3).

**Table 3:** Adjusted odds ratio (AOR) and 95% confidence interval (CI) of oral squamous cell carcinoma (OSCC) associated with *HMGB1* genotypic frequencies

Variables	Controls (n=1,200)(%)	Patients (n=772)(%)	OR (95% CI) †	AOR (95% CI) ‡
**rs1412125**				
TT	649 (54.1%)	438 (56.7%)	1.00	1.00
TC	457 (38.1%)	274 (35.5%)	0.888 (0.733-1.077)	0.993 (0.772-1.277)
CC	94 (7.8%)	60 (7.8%)	0.946 (0.669-1.337)	0.975 (0.622-1.527)
TC+CC	551 (45.9%)	334 (43.3%)	0.898 (0.749-1.078)	0.990 (0.780-1.255)
**rs2249825**				
CC	852 (71.0%)	573 (74.2%)	1.00	1.00
CG	316 (26.3%)	183 (23.7%)	0.861 (0.698-1.063)	0.848 (0.644-1.116)
GG	32 (2.7%)	16 (2.1%)	0.743 (0.404-1.367)	0.512 (0.243-1.077)
CG+GG	348 (29.0%)	199 (25.8%)	0.850 (0.693-1.043)	0.807 (0.619-1.053)
**rs1045411**				
CC	723 (60.3%)	507 (65.7%)	1.00	1.00
CT	411 (34.3%)	226 (29.3%)	**0.784 (0.643-0.956)**	**0.754 (0.582-0.978)**
TT	66 (5.5%)	39 (5.1%)	0.843 (0.558-1.272)	0.962 (0.565-1.636)
CT+TT	477 (39.8%)	265 (34.3%)	**0.792 (0.656-0.956)**	**0.778 (0.609-0.995)**
**rs1360485**				
TT	682 (56.8%)	452 (58.5%)	1.00	1.00
TC	440 (36.7%)	273 (35.4%)	0.936 (0.772-1.135)	0.926 (0.719-1.919)
CC	78 (6.5%)	47 (6.1%)	0.909 (0.621-1.331)	0.998 (0.609-1.636)
TC+CC	518 (43.2%)	320 (41.5%)	0.932 (0.776-1.119)	0.936 (0.737-1.190)

^†^The odds ratio (OR) with their 95% confidence intervals were estimated bylogistic regression models.

^‡^The adjusted odds ratio (AOR) with their 95% confidence intervals were estimated by multiple logistic regression models after controlling for betel quid chewing, alcohol and tobacco consumption.

### Functional effect of rs1045411 on *HMGB1* transcript stability

**Figure 1:**
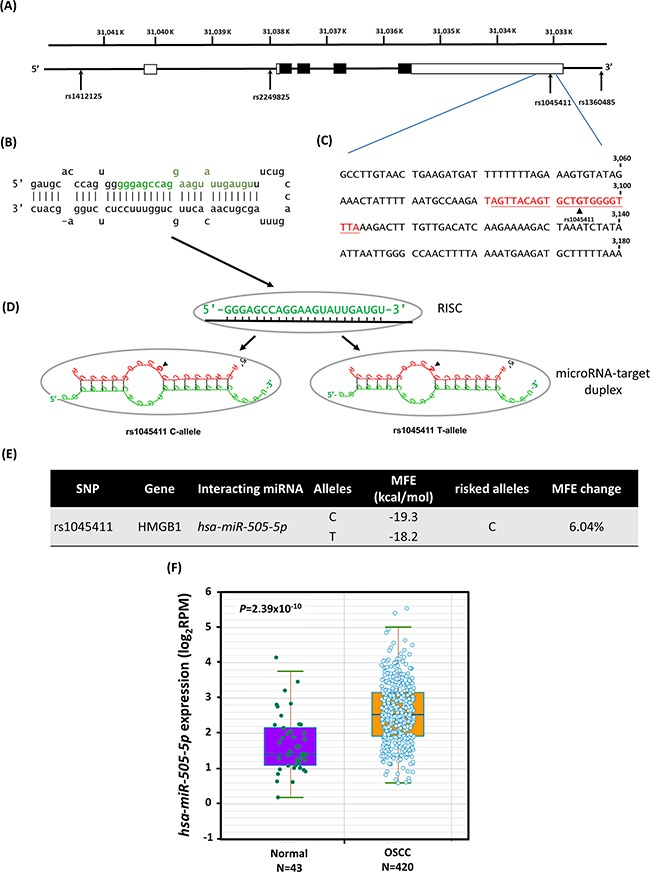
Binding site polymorphism for SNP rs1045411 [G/A] in the human *HMGB1* 3’-UTR mRNA with a microRNA *hsa-miR-505-5p* binding site, decreases OSCC susceptibility among the Taiwan OSCC population **(A)** Exons of HMBG1 are shown as filled boxes along the chromosome (chr.13, reference genome GRCh37.p13). **(B)** The stem-loop portion of the miRNA-miRNA duplex structure of pre-miRNAs (*hsa-miR-505;* miRBase ID: MI0003190) was identified by miRNA target prediction using the MicroRNA.org resource. The *hsa-miR-505-5p* sequence is marked in green. **(C)** Sequence of the human *HMBG1* 3’-UTR region; the number shows the positions of the mRNA (NM_002128.5). The predicted *hsa-miR-505-5p* binding site of SNP rs1045411 is highlighted in color red. **(D)** Models of the miRNA-target duplex were determined using the RNAhybrid web tool on Bielefeld Bioinformatics Server. RISC, RNA-induced silencing complex; arrows indicate the location of rs1045411. **(E)** The SNP rs1045411 C-allele reduces the free binding energy (MFE, minimum free energy; change: 6.04%). **(F)** Boxplot chart showing the differential expressions of miRNA *hsa-miR-505-5p* in 420 OSCC patients and 43 normal controls, as taken from the Pan-Cancer dataset.

All of the subjects were genotyped for the four SNPs, which were selected to cover (*r^2^* ≥ 0.70) most of the SNPs located in a 12 kb region that includes the *HMGB1* gene (8 kb), its promoter (2 kb) and the 3’-UTR (2 kb) (Figure 1A). The 3’-untranslated region (3’-UTR) of the *HMGB1* gene covers 2.0 kb and might be among the region most sensitive to microRNA (miRNA) epigenetic regulation [31]. An miRNA, *hsa-miR-505-5p* [32, 33] (miRBase [34] ID: MI0003190, Figure 1B), shares binding site complementarily with rs1045411 in the 3’-UTR (Figures 1C and 1D). In addition, the OSCC-associated risk [C]-allele creates a slight kink in the *HMGB1* mRNA structure compared to the [T]-allele, which results in a less negative free-energy state, and less stable hybridization [MFE (minimum free energy) change: 6.04%, from -19.3 kcal/mol to -18.2 kcal/mol] (Figure 1E). Furthermore, starBase [35] analysis revealed an 1.86-fold (*P*=2.39×10^-10^) increase in *hsa-miRNA-505-5p* expression in 420 OSCC patients and 43 subjects from the Pan-Cancer dataset [36] (Figure 1F). Accordingly, the location of the 3’-UTR SNP rs1045411 [C-allele] alter *HMGB1* mRNA stability and increase susceptibility to OSCC (Figure 1E).

### Synergistic effects of genetic variants and betel quid chewing behavior with smoking

The habit of betel quid chewing widespread in Taiwan, and chewers are often smokers. Thus, we analyzed the synergistic effects of cigarette smoking and betel chewing combined with the four SNPs in the OSCC patients. Among the 1,322 smokers in our study, the estimated AORs of the four selected *HMGB1* polymorphisms were elevated in individuals who were both betel quid chewers and smokers (10.071-10.659-fold) compared to abstainers (Table 4). However, the risk of OSCC tended to decline among those who had quit betel quid chewing, suggesting that the four selected *HMGB1* variants are associated with betel quid chewing and OSCC susceptibility in cigarette smokers (Table 4).

**Table 4:** Associations of the combined effect of *HMGB1* gene polymorphisms and betel quid chewing with the susceptibility to oral squamous cell carcinoma (OSCC) among 1,322 smokers

Variable	Controls(n=636) (%)	Patients(n=686) (%)	OR (95% CI) †	AOR (95% CI) ‡
**rs1412125**				
TT genotype & non-betel quid chewing ^a^	231 (36.3%)	59 (8.6%)	1.00	1.00
TC or CC genotype or betel quid chewing ^b^	331 (52.0%)	375 (54.7%)	**4.436 (3.215-6.120)**	**3.604 (2.534-5.126)**
TC or CC genotype with betel quid chewing ^c^	74 (11.6%)	252 (36.7%)	**13.333 (9.065-19.611)**	**10.164 (6.688-15.446)**
**rs2249825**				
CC genotype & non-betel quid chewing ^a^	314 (49.4%)	82 (12.0%)	1.00	1.00
CG or GG genotype or betel quid chewing ^b^	269 (42.3%)	446 (65.0%)	**6.349 (4.768-8.454)**	**5.465 (3.982-7.501)**
CG or GG genotype with betel quid chewing ^c^	53 (8.3%)	158 (23.0%)	**11.416 (7.692-16.942)**	**10.026 (6.505-15.453)**
**rs1045411**				
CC genotype & non-betel quid chewing ^a^	280 (44.0%)	71 (10.3%)	1.00	1.00
CT or TT genotype or betel quid chewing ^b^	288 (45.3%)	403 (58.7%)	**5.518 (4.083-7.458)**	**4.877 (3.494-6.809)**
CT or TT genotype with betel quid chewing ^c^	68 (10.7%)	212 (30.9%)	**12.295 (8.430-17.932)**	**10.071 (6.670-15.208)**
**rs1360485**				
TT genotype & non-betel quid chewing ^a^	262 (41.2%)	66 (9.6%)	1.00	1.00
TC or CC genotype or betel quid chewing ^b^	301 (47.3%)	371 (54.1%)	**4.893 (3.589-6.670)**	**4.383 (3.111-6.176)**
TC or CC genotype with betel quid chewing ^c^	73 (11.5%)	249 (36.3%)	**13.540 (9.303-19.709)**	**10.659 (7.073-16.065)**

^†^The odds ratio (OR) with their 95% confidence intervals were estimated bylogistic regression models.

^‡^The adjusted odds ratio (AOR) with their 95% confidence intervals were estimated by multiple logistic regression models after controlling for betel quid chewing, alcohol and tobacco consumption.

^a.^Individuals are homozygous for ancestral allele but without betel quid chewing.

^b.^Individuals with either at least one polymorphic allele or betel quid chewing.

^c.^Individuals with both at least one polymorphic allele and betel quid chewing.

### Haplotypes analysis for the *HMGB1* gene

**Figure 2:**
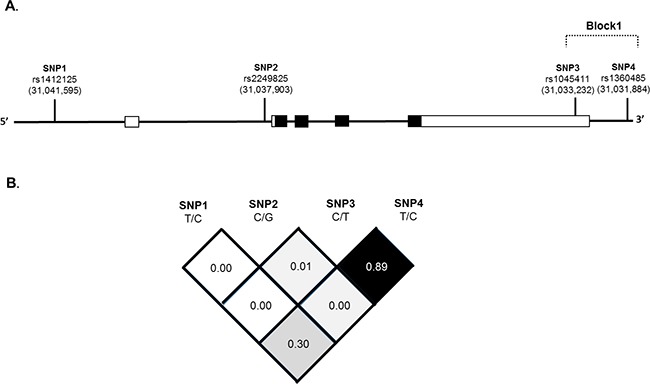
The *HMGB1* gene, locations of the genotyped variants and their pairwise linkage disequilibrium patterns Schematic presentation of the *HMGB1* (gene ID: 3146), **(A)** indicating the locations of the analyzed variants (rs1412125, rs2249825, rs1045411 and rs1360485). SNPs within and around the *HMGB1* gene were plotted against the chromosomal positions chr.13: 29,928,000 to 29,940,000 (HapMap version 3 release 27). Exons, introns and untranslated regions are shown by filled boxes, unfilled and thin lines from 5’- to 3’- end of this gene, respectively. **(B)** In the LD map SNPs-pairwise correlation coefficients, r^2^, in East Asian population (HCB+JPT) are shown in the square black when D’=1.0, while the square is white when D’=0. A “Confidence intervals” conventional gray scale is used to display LD were shown in black through gray (color intensity decreasing with decrease D’ value) generated using Haploview version 4.2.

*HMGB1* polymorphisms were further characterized using linkage disequilibrium (LD) and haplotype analyses. LD was determined pairwise among all 4 SNPs and the haplotype structure of the *HMGB1* gene was analyzed (D’ and *r^2^*) according to 1000 Genomes Project data [37] for the East Asian population (CHB+JPT, Figure 2). Haplotype blocks divided by the D’ confidence interval method with a D’ value of 95% CI 0.70-0.98 in adjacent SNPs was classified as the same haplotype block. One LD block was detected by Solid Spine [38], a haplotype phasing technique. Block1 (3 kb) consisted of two closely selective SNPs showing strong linkage, rs1045411 and rs1360485, in the 3’-UTR of *HMGB1* (Figure 2).

Furthermore, a haplotype-based association study was performed to show association between *HMGB1* haplotypes and OSCC risk (Table 5). Block1 of the 3’-UTR SNPs constituted virtually four haplotypes of approximately equal frequencies in the control subjects (75.0%, 22.5%, 2.3% and 0.1%). In contrast, one haplotype in the OSCC cases, “C-C”, was associated with increased susceptibility to OSCC (AOR=1.808; 95% CI, 1.257-2.601, *P*=0.001) compared with the most common “C-T” haplotype (Table 5).

**Table 5:** The estimated haplotype frequencies of four examined polymorphisms in *HMGB1* gene and the corresponding risk for oral squamous cell carcinoma (OSCC)

Haplotype block		Controls (n=2,400)(%)	Patients (n=1,544)(%)	OR (95% CI)	*P* value
rs1045411 C/T	rs1360485 T/C				
C	T	1,801 (75.0%)	1,174 (76.0%)	1.00	
T	C	540 (22.5 %)	301 (19.5%)	0.855 (0.729-1.002)	0.054
C	C	56 (2.3%)	66 (4.3%)	**1.808 (1.257-2.601)**	**0.001**
T	T	3 (0.1%)	3 (0.2%)	1.534 (0.309-7.613)	0.598

### The effects of rs1412125 and rs2249825 on *HMGB1* transcriptional regulation

**Figure 3:**
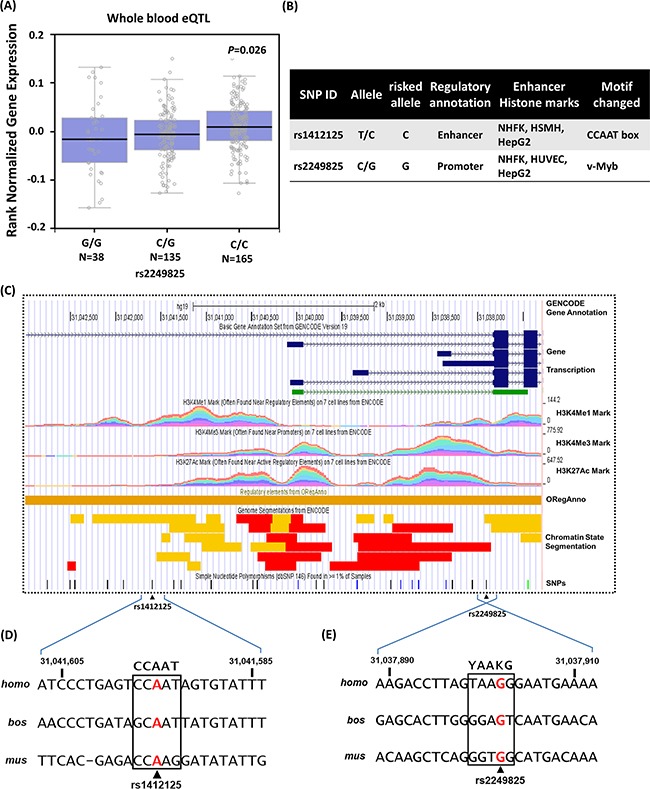
Binding sites polymorphism for SNPs rs1412125 [T/C] and rs2249825 [C/G] provide transcriptional control to regulate expression of the human *HMGB1* gene (NM_002128.5) **(A)** Expression quantitative trait locus (eQTL) association between rs2249825 and *HMGB1* expression in whole blood (GTEx data set). The numbers in parentheses indicate the number of cases. **(B)** Regulatory annotation of variants from ENCODE data showing evidence of enhancer elements coinciding with rs1412125 and rs2249825 in many different cell types. In addition, the CCAAT/Enhancer box and v-Myb motif are predicted to be affected. **(C)** Expanded view of ENCODE data for the *HMGB1* promoter and intron 1, containing rs1412125 and rs2249825, in the GRCh37/hg19 assembly using the UCSC genome browser. In Gene Transcription track, shown color blue and green indicated coding and non-coding *HMGB1* proteins, respectively. H3K4Me1, H3K4Me3, and H3K27Ac tracks show the genome-wide levels of enrichment of the mono-methylation of lysine 4, tri-methylation of lysine 4, and acetylation of lysine 27 of the H3 histone protein, as determined by the ChIP-seq assays. These levels are thought to be associated with promoter and enhancer regions. Chromatin State Segmentation track displays chromatin state segmentations by integrating ChIP-seq data using a Hidden Markov Model for H1-hESC (embryonic stem cells; color yellow), HepG2 (hepatocellular carcinoma cells; color orange), HUVEC (umbilical vein endothelial cells; color light blue), HMEC mammary epithelial cells, HSMM (skeletal muscle myoblasts; color green), NHEK (epidermal keratinocytes; color purple), and NHLF (lung fibroblasts; color red). The chromatin state regions predicted for promoters and enhancer are highlighted by red and yellow, respectively. ORegAnno track shows transcription factor binding sites. Enhancers are typically common near transcription start sites and may be associated with promoter regions. **(D)** Sequence of the human *HMGB1* promoter region and rs1412125. Consensus residues of the CCAAT/Enhancer box core consensus sequence are indicated in bold fonts. **(E)** Reversed sequence of the human *HMGB1* intron region and rs2249825. Consensus residues of the core v-Myb consensus sequence are indicated in bold font, where Y denotes C or T and K denotes G or T.

As a preliminary assessment of the putative functional role of these SNPs, we investigated whether rs1412125 and rs2249825 are associated with differential expression of *HMGB1*, According to the Genome-Tissue Expression (GTEx) database [39], statistically significant down-regulation of *HMGB1* mRNA expression with rs2249825 variant genotype (C/G+G/G) compared with the wild-type homozygous genotype (C/C, *P*=0.026, Figure 3A) is observed in whole blood. Moreover, OSCC-risk-associated environmental factors were further explored by examining functional annotation in the Encyclopedia of DNA elements (ENCODE) data [40] for sites in the 3,500-bp promoter and intron 1 sequences (Figure 3B and 3C). We determined that rs1412125 and rs2249825 are situated at a site for transcription factor binding, with histone modification patterns and DNA hyposensitivity characterized in the promoter or enhancers in several cell type (Figure 3C). The risk allele [T] of rs1412125 in located in the core of a CCAAT/Enhancer box [41] (Figure 3D), which is one of the most common elements in eukaryotic promoter. The effect of rs2249825 may be attributed to the suboptimal avian myeloblastosis viral oncogene homolog-like 2 (v-Myb) binding site [42] (Figure 3E), with the consensus motif of (C/T)AA(G/T)G surrounding intron 1 of the predicted transcriptional start site of human *HMGB1* gene (Figure 3B), which enables modulating of initiation rates in response to transcriptional status. Accordingly, these promoter polymorphisms in the CCAAT/Enhancer box and v-Myb binding site might collaborate in the regulation of *HMGB1* expression.

## DISCUSSION

Cancer is the result of a series of genetic or epigenetic regulations, and environmental risk factors may contribute to the development and progression of tumors [21, 43]. Thus, in-depth understanding of the mechanisms underlying carcinogenesis is needed to characterize genetic alterations linked to OSCC development [44]. Such information may provide relevant strategies for prevention, for the surveillance of patients at risk, and for early detection to reduce morbidity and mortality from OSCC [45]. Several studies have suggested that genetic alternations harbored on chromosome 13q12 are involved in multiple tumor types [46–50] and this locus is highly susceptibility to genomic instability in OSCC [51]. Recent studies have reported that *HMGB1* polymorphism is associated with susceptibility to several carcinomas including breast cancer [52], melanoma [53], gastric cancer [54] and colorectal cancer [55]. Loss of *HMGB1* increase DNA damage due to decreases in DNA chaperone efficiency in response chemotherapy, cell stress, and environmental risk factors [56]. Our data revealed an increased risk of OSCC among patients with the *HMGB1* polymorphism rs1045411 C/C compared with those with the C/T or C/T+T/T genotype (Table 3). We present additional evidence for a role for *HMGB1* in OSCC by showing a significant interaction between betel quid chewing and at least one polymorphic allele of four *HMGB1* SNPs in individuals, higher incidence of OSCC with smoking behavior (Table 4).

The present study confirms the association of the clinically examined rs1045411 and rs1360485 haplotype in the *HMGB1* 3’-UTR region with OSCC risk most likely because a putative *miRNA-505* binding site (Figure 1). We confirmed that these two *HMGB1* 3’-UTR variants are synergistically involved in the etiopathogenesis of OSCC in a haplotype-specific fashion rather than as single genetic variants (Table 5). Therefore, an SNP haplotype corresponding to the RNA bulge region may effects the binding strength of specific miRNA/target duplexes, resulting in low minimum free energy and modulating mRNA stability (Figure 1). Although directly testing this hypothesis is beyond the scope of the current study, evidence suggests that mRNA stability is associated with susceptibility to OSCC, with *HMGB1* downregulation being associated with an increased susceptibility to OSCC. Previous studies demonstrate that *miRNA-505* acts as tumor suppressor in endometrial carcinoma [57]. Matamala et al. also reported that circulating *miRNA-505* was significantly overexpressed in breast cancer patients [58]. Moreover, *miRNA-505* suppresses proliferation and invasion in hepatoma cells by directly targeting *HMGB1* has been previously reported [59]. Hence, the present analyses increased our understanding of naturally occurring *HMGB1* variants, a lesion category that, while not infrequent, has been relatively neglected in terms of elucidation of the underlying pathogenic mechanisms. *HMGB1* expression is considered as a novel and independent predictor of decreased disease-free survival of patients with prostate cancer [25, 60], and it may also be used as a novel and independent predictor of prognosis for OSCC patients.

Converging lines of evidence support that OSCC development is a complicated process regulated by both environmental and genetic factors. In the present study, we estimated the magnitude of the statistical interaction between two measurable exposures with respect to a given outcome with *HMGB1* genetic variants and found that the use of betel quid with smoking conferred a higher susceptibility to OSCC (Table 4). Strikingly, polymorphisms altering DNA chaperone activity, which maintains nuclear homeostasis, may lead to synergistic effects in areca nut carcinogen-induced OSCC risk [61]. However, the effects of environmental risks on predisposition to OSCC may be underestimated because of the inability to quantitatively access the degree of betel quid chewing and DNA chaperone gene variations with smoking. Nevertheless, this study provides comprehensive evidence for the association of several genetic variation in the DNA-chaperon *HMGB1* gene and multiple environmental exposures in OSCC development [62]. However, there are several limitations in our investigation. One of the limitations is that information on exposures of carcinogens is dichotomized into ‘‘ever-user’’ versus ‘‘never-user.’’ More detailed analysis based on amount on exposures of carcinogens should be performed. Furthermore, the molecular functional role in the *HMGB1* 3’UTR mRNA with a *miRNA-505*-binding site of OSCC requires further investigation.

To our knowledge, there are no previous studies of the effects of gene-environment interactions on DNA chaperon gene polymorphisms and betel-chewing habits in oral tumorigenesis. Nonetheless, the co-effects of genetic and environmental factors interact to facilitate OSCC development. Overall, we present a possible role for *HMGB1* variants and provide deeper insights into naturally occurring haplotype-based variants. Furthermore, characterizing the molecular basis of polymorphisms in cancer provides insight into tumorigenesis. Accurate biomarkers for such types of variants are required for developing optimal therapeutic approaches that will eventually ameliorate the clinical phenotype in patients harboring the corresponding lesions.

## MATERIALS AND METHODS

### Description of the participants

This study recruited 772 male OSCC patients between 2008 and 2015 at Chung Shan Medical University Hospital, Taiwan. Clinically information including TNM staging, primary tumor size, lymph node involvement, and histologic grade was examined according to American Joint Committee on Cancer (AJCC) classification [63, 64]. Subjects with neither self-reported history of cancer of any site nor oral precancerous disease such as oral submucous fibrosis, leukoplakia, erythroplakia, or verrucous hyperplasia were selected to the control group from Taiwan Biobank. Before the study began, approval was obtained from the Institutional Review Board of Chung Shan Medical University Hospital, and informed written consent was obtained from each individual.

### SNPs selection and genotyping

Genomic DNA was isolated from peripheral blood using the QIAamp DNA blood mini kit as described in detail previously [65, 66]. The final preparation was stored at -20°C, quantified by optical density measurement at 260 nm and used as the template for polymerase chain reaction (PCR). Genotyping of four *HMGB1* SNPs (rs1412125, rs2249825, rs1045411, and rs1360485; Table 1) with minor allele frequencies >5% in the HapMap Chinese Han Beijing (CHB) population was performed using the TaqMan SNP genotyping assay (Applied Biosystems, Foster City, CA, USA) [67]. Allele frequencies were determined by the ABI SDS software.

### Bioinformatics analysis

Several semi-automated bioinformatics tools were applied to assess whether SNPs or their linked genetic variants are associated with a putative function that might affect patient outcomes. HaploReg [68] v4 and the GTEx database [39] from the ENCODE project [69] were used to identify the regulatory potential of candidate functional variants. Particular sites of interest were examined including transcription factor (TF)-ChIP signals, DNase peaks, DNase footprints and predicted DNA sequence motifs for TFs. The GTEx data were used to identify correlations between SNPs and whole blood-specific gene expression levels. The publically available cBioPortal for Cancer Genomics [70] and UCSC Cancer Genomics Browser [71] for OSCC were utilized to analyse *HMGB1* gene expression, enhancer modulation, molecular features, and clinical outcomes.

### Characteristics of miRNA candidates

We predicted targets using the web-based tools RNAhybrid on BiBiServ2 (http://bibiserv.techfak.uni-bielefeld.de/rnahybrid). RNAhybrid [72] determines the most energetically favorable hybridization patterns using the MFE of two RNA fragments with different lengths, i.e. long (3’-UTR of *HMGB1*) and short (mature miRNA sequences). The parameters used in the analysis were as follows: number of hits per target, 3 nucleotides, maximum mismatch size, 1 nucleotide, overhangs 2 nucleotides. The MFE considered for each miRNA/target duplex was higher than -15 kcal/mole as assessed for a perfect match between the mature miRNA and its target.

### Statistical analysis

The Mann–Whitney U test and Fisher's exact test were used to compare differences in the distribution of age and demographic characteristics between the controls and OSCC patients. ORs with 95% CIs were estimated using logistic regression models. AORs with 95% CIs were used to assess association between genotype frequencies with OSCC risk and clinical factors. *P* values less than 0.05 were considered significant. The data were analysed with SPSS 12.0 statistical software (SPSS Inc., Chicago, IL, USA).
